# Ultrathin acoustic metamaterial as super absorber for broadband low-frequency underwater sound

**DOI:** 10.1038/s41598-023-34993-0

**Published:** 2023-05-17

**Authors:** Xindong Zhou, Xiaochen Wang, Fengxian Xin

**Affiliations:** 1 State Key Laboratory for Strength and Vibration of Mechanical Structures, Xi’an Jiaotong University, Xi’an, 710049 People’s Republic of China; 2 MOE Key Laboratory for Multifunctional Materials and Structures, Xi’an Jiaotong University, Xi’an, 710049 People’s Republic of China

**Keywords:** Engineering, Applied physics

## Abstract

In this work, an ultrathin acoustic metamaterial formed by space-coiled water channels with a rubber coating is proposed for underwater sound absorption. The proposed metamaterial achieves perfect sound absorption ($\alpha$ > 0.99) at 181 Hz, which has a deep subwavelength thickness ($\lambda {/}162$). The theoretical prediction is consistent with the numerical simulation, which demonstrate the broadband low-frequency sound absorption performance of the proposed super absorber. The introduction of rubber coating leads to a significant decrease of the effective sound speed in the water channel, resulting in the phenomenon of slow-sound propagation. From the perspective of numerical simulations and acoustic impedance analysis, it is proved that the rubber coating on the channel boundary causes slow-sound propagation with inherent dissipation, which is the key to meet the impedance matching condition and achieve perfect low-frequency sound absorption. Parametric studies are also carried out to investigate the effect of specific structural and material parameters on sound absorption. By tailoring key geometric parameters, an ultra-broadband underwater sound absorber is constructed, with a perfect absorption range of 365–900 Hz and a deep subwavelength thickness of 33 mm. This work paves a new way for designing underwater acoustic metamaterials and controlling underwater acoustic waves.

## Introduction

Underwater sound absorption is critical for underwater applications such as acoustic stealth^1^. To be an underwater acoustic material, it should meet two requirements, i.e., its acoustic impedance should match with the water and have a high loss damping factor^2^. Viscoelastic polymers such as rubber and polyurethane can well meet these two requirements, so they are widely used in underwater sound absorption materials^3^. Using viscoelastic polymers as the matrix material, various underwater anechoic layers with excellent acoustic performance can be designed, such as cavity type^4,5^ and particle-filled type^6^, etc. These traditional underwater acoustic materials often cannot withstand high hydrostatic pressure^7^, which limits the development of their applications. In addition, researchers have designed a series of other materials and structures for underwater sound absorption, such as porous foam materials^8–11^ and locally resonant acoustic materials^12–23^. These materials have achieved good sound absorption performance at medium and high frequencies.

In recent years, with the development of marine equipment, the requirements for sound absorption performance have been continuously improved, and it is developing towards subkilohertz frequency broadband underwater sound absorption in subwavelength scale. Traditional underwater sound-absorbing materials are difficult to meet these requirements. The recent emergence of a series of airborne and waterborne acoustic metamaterials offers the possibility to address this critical problem^24–35^. In the field of airborne sound, sound absorption below kilohertz frequencies is commonly achieved by constructing Helmholtz resonance structures^34,35^, space-coiled structures^25,26^ and membrane structures^24^. Compared with air sound absorption, it is more difficult to achieve effective underwater sound absorption due to the longer wavelength of underwater sound and the lower viscosity of water. On the one hand, the waterborne sound wavelength is about 5 times of airborne sound wavelength at the same frequency, which makes it more difficult to control waterborne sound, especially the low-frequency waterborne sound. On the other hand, the kinematic viscosity of water is much smaller, about 1/15 of that of air, which weakens the viscous effect of water in sound energy dissipation. Therefore, ordinary airborne sound absorbers are not applicable to the underwater environment. Underwater acoustic metamaterials are usually based on viscoelastic matrices such as rubber to construct local resonance structures, impedance matching structures^28,32^ and pentamode metamaterials^31^ for low-frequency sound absorption. Ultrathin composite metasurfaces based on pentamode metamaterials exhibit good underwater sound absorption^31^. The application of impedance-matched composite enables underwater sound absorption at several kilohertz frequencies^32^. The research on cavity-type anechoic coatings under hydrostatic pressure has been further developed and refined^33^. However, it is still a great challenge for researchers to design underwater acoustic metamaterials with subwavelength broadband low-frequency sound absorption.

In this paper, an ultrathin acoustic metamaterial with deep subwavelength thickness is proposed as super absorber for broadband low-frequency underwater sound. The unit cell of the proposed metamaterial consists of a perforated plate and a space-coiled water channel with a rubber coating attached to the channel wall. A theoretical model and a numerical model are established to calculate the sound absorption properties of the metamaterial, which agree well with each other. Based on the sound pressure distribution and energy dissipation obtained from numerical simulation and acoustic impedance analysis, the reasons for the slow-sound propagation and the mechanism of the excellent sound absorption performance are analyzed. The influence of key parameters of the metamaterial on the effective sound speed and sound absorption coefficient is discussed. On this basis, an ultrathin acoustic metamaterial with parallel hybrid units is designed for broadband low-frequency underwater sound absorption.

## Theoretical model

**Figure 1:**
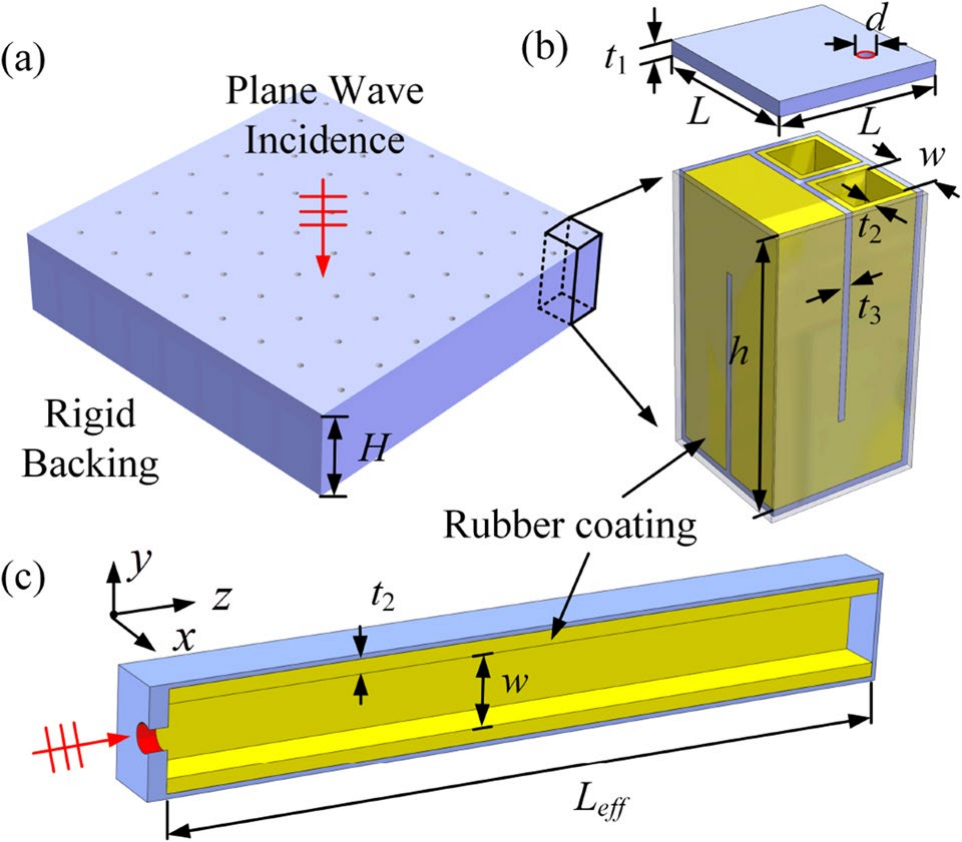
(**a**) Geometry of the proposed acoustic metamaterial with thickness $H$. Plane incident waves are considered to be incident on the metamaterial. (**b**) A representative unit of the proposed metamaterial with width $L$, consisting of a perforated panel (thickness $t_{1}$, perforation diameter $d$, perforation ratio $\sigma = \pi d^{2} /4L^{2}$) and a space-coiled water channel (channel width $w$, height $h$) with a rubber coating (yellow area, thickness $t_{2}$) on the channel wall. The thickness of the walls is $t_{3}$. (**c**) Equivalent model of the proposed metamaterial with straight water channel (effective channel length $L_{eff} = V/w^{2}$, where $V$ is the volume of the water channel).

The schematic diagram of the proposed ultrathin acoustic metamaterial with periodically arranged units is shown in Fig. 1a. Each unit cell consists of a perforated panel and a space-coiled water channel attached with a rubber coating on the channel wall, as shown in Fig. 1b. To clearly show the internal structure of the metamaterial, the perforated plate in Fig. 1b is moved upward. The rubber coating is attached to the space-coiled channel wall and the lower surface of the perforated plate. The space-coiled channel design is applied to reduce the thickness of the sound absorber without sacrificing the sound absorption performance. The perforation of the perforated panel and the water channel can form a Helmholtz resonant cavity. The introduction of the rubber coating leads to the quasi-Helmholtz resonance, which can be used to absorb low-frequency underwater sound^36^.

An equivalent model for the underwater sound absorption of the acoustic metamaterial is established, which simplifies the space-coiled channel into a straight channel, as shown in Fig. 1c. The effective channel length $L_{eff}$ and the rubber coating thickness $t_{2}$ are the key parameters to determine the acoustic performance of the metamaterial, which remain unchanged after simplification. This ensures that the simplification of the equivalent model is reasonable. In the equivalent model, the surface area of the perforated panel appears to be reduced, which would result in a lower surface acoustic impedance of the equivalent model than that of the metamaterial. In order to maintain the accuracy of the equivalent model, the actual surface area (L*L) of the perforated panel is used in theoretical analysis. The perforated panel and channel wall are considered to be made of steel, and these steel parts are assumed to be acoustically rigid in the theoretical analysis (see Supplementary materials for details of the validation of this assumption). Due to the great mechanical strength of steel compared to rubber and water, the effect of the elastic behavior of steel on the acoustic performance is negligible in theoretical model. In the following, theoretical and numerical models are established to study the underwater sound absorption performance of the proposed metamaterial.

The sound absorption coefficient of the proposed acoustic metamaterial with rigid backing can be calculated by

1 $$ \alpha = 1 - R^{2} = 1 - \left| {\frac{{Z_{s} - \rho_{0} c_{0} }}{{Z_{s} + \rho_{0} c_{0} }}} \right|^{2} $$

where $R$ is the reflection coefficient, $\rho_{0}$ and $c_{0}$ are the density and sound speed of water. $Z_{s} = Z_{p} + Z_{c}$ is the surface acoustic impedance of the metamaterial, where $Z_{p}$ and $Z_{c}$ are the acoustic impedance of the perforated panel and the water channel, respectively.

The acoustic impedance of the perforated panel is^36^

2 $$ Z_{p} = \frac{{i\omega \rho_{0} t_{1} }}{\sigma }\left[ {1 - \frac{{2J_{1} (y\sqrt { - j} )}}{{(y\sqrt { - j} )J_{0} (y\sqrt { - j} )}}} \right]^{ - 1} + \frac{{\sqrt 2 \eta_{0} y}}{\sigma d} + j\frac{{0.85\omega \rho_{0} d}}{\sigma } $$

where $J_{n}$ is the first kind Bessel function of $n$-th order, and $y = d\sqrt {\omega \rho_{0} /4\eta_{0} }$ is $\sqrt 2 /2$ times the ratio of the perforation diameter to the viscous boundary layer thickness. $j$ is the imaginary unit, $\eta_{0} = 1.01 \times 10^{ - 3} {\text{ Pa}} \cdot {\text{s}}$ is the dynamic viscosity of water.

The acoustic impedance of the water channel is

3 $$ Z_{c} = - j\xi \rho_{0} c_{z} \cot \left( {\omega L_{eff} /c_{z} } \right) $$

where $\xi = w^{2} /L^{2}$ is an area modification factor that takes into account the cross-sectional discontinuity between the water channel and the actual surface area (L*L). $\omega$ is the angular frequency with $f$ being the frequency of incident acoustic wave. $c_{z}$ is the effective sound speed along the z-direction on the water channel. To determine $c_{z}$, the influence of the non-rigid boundary condition of the water channel needs to be considered, which is derived from the vibration of rubber coating. Only the compression vibration of the rubber coating is considered here, and the shear vibration of the rubber coating is weak and can be ignored. The non-rigid boundary condition of the water channel can be expressed as

4 $$ u_{r} = p/Z_{r} $$

where $u_{r}$ and $p$ are the normal particle velocity and sound pressure at the boundary of the water channel, respectively. $Z_{r} = - jZ_{1} /k_{r} t_{r}$ is the normal acoustic impedance of rubber coating, where $Z_{1} = \rho_{r} c_{r}$ is the characteristic acoustic impendence of rubber, with $\rho_{r}$ and $c_{r} = \sqrt {K_{r} /\rho_{r} }$ being the density and longitudinal wave speed of rubber, respectively. $K_{r} = (1 + j\eta_{r} )\frac{{E_{r} (1 - \mu_{r} )}}{{(1 + \mu_{r} )(1 - 2\mu_{r} )}}$ is the complex bulk modulus of rubber, with $\eta_{r}$, $E_{r}$ and $\mu_{r}$ being the isotropic loss factor, Young’s modulus and Poisson’s ratio of rubber. The isotropic loss factor is set as a frequency independent parameter that can be adjusted as needed^20,22,28,37,38^. $k_{r} = \omega /c_{r}$ is the acoustic wavenumber in rubber. $t_{r} = S_{r} /l_{r} = \left[ {(w + 2t_{2} )^{2} - w^{2} } \right]/4w$ is the effective thickness of rubber coating, where $S_{r}$ is the effective area of the rubber in the section and $l_{r}$ is the inner perimeter of the water channel section. By directly substituting $Z_{s} = Z_{r}$ into Eq. (1), the sound absorption coefficient of homogeneous rubber with rigid backing can be obtained.

The three-dimensional wave equation on the water channel can be solved by using the non-rigid boundary condition Eq. (4). And then the effective sound speed $c_{z}$ on the water channel can be calculated as^39^

5 $$ c_{z} = c_{0} /\sqrt {1 + 4t_{r} \frac{1}{w}\frac{{\rho_{0} c_{0}^{2} }}{{\rho_{r} c_{r}^{2} }}} $$

where $c_{z}$ is a frequency independent constant as a function of $(t_{2} ,w,\eta_{r} )$. When the thickness of rubber coating $t_{2}$ is equal to 0, we can get $c_{z} = c_{0}$. Substituting Eq. (5) into Eq. (3), the acoustic impedance of the water channel can be calculated, and then the sound absorption coefficient of the metamaterial can be predicted theoretically.

## Finite element model

**Figure 2:**
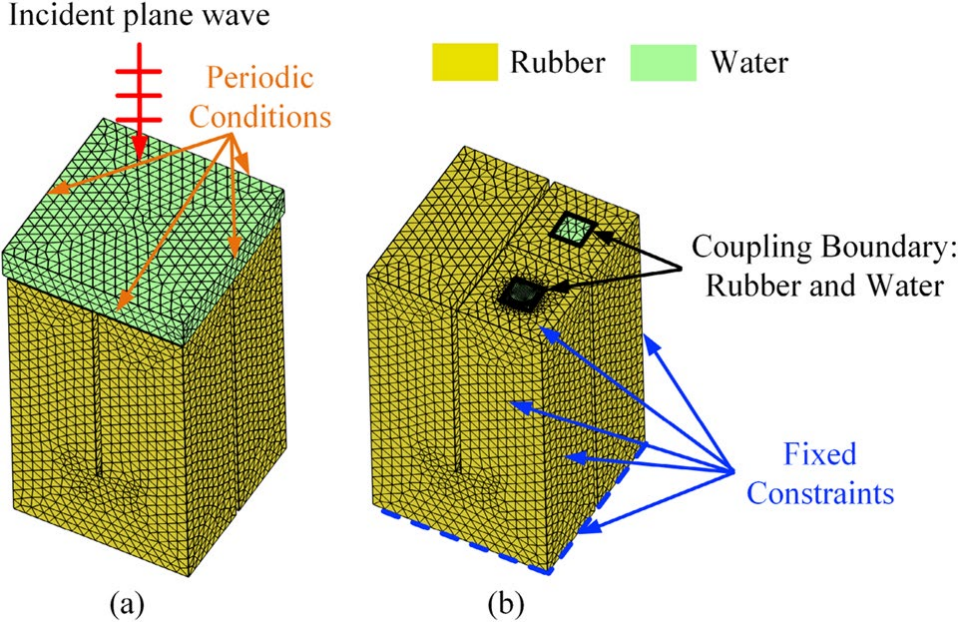
Finite element model for the sound absorption of the proposed metamaterial. The yellow and green sections represent the rubber and water areas, respectively.

A numerical model based on the finite element method (FEM) is established in COMSOL Multiphysics software to validate the theoretical model, as shown in Fig. 2. Due to the periodicity of the proposed metamaterial, only one unit cell needs to be modeled, as shown in Fig. 2a. In the finite element model, the solid mechanics module is selected for the rubber region displayed in yellow, and the thermo-viscous acoustic module is selected for the water region displayed in green. The viscous dissipation and thermal loss are considered in the thermo-viscous acoustic module. An additional water region is set above the metamaterial surface to simulate the acoustic incident field, and plane wave incidence is set on the top surface of the acoustic incident field. Two pairs of periodic conditions are applied to both sides of the acoustic incident field to model this periodic acoustic metamaterial. As shown in Fig. 2b, due to the continuity of sound pressure and particle vibration velocity at the water-rubber coupling interfaces, the acoustic-structure interaction boundaries are set at these coupling interfaces. The steel parts are considered acoustically rigid, so the interfaces between steel and rubber are set as fixed constraints, while the interfaces between steel and water are set as sound hard boundaries. The bottom surface of the metamaterial is set as a fixed constraint considering the rigid backing. Although the acoustically rigid steel assumption is somewhat idealized, in the Supplementary materials we have developed a finite element model considering steel parts as elastic bodies. The results of this elastic finite element model agree well with those of the rigid finite element model, confirming that the rigid finite element model can accurately predict the acoustic properties of the proposed metamaterial within the frequency band considered here (see Supplementary materials for details of the validation of this assumption).

The surface acoustic impedance can be calculated from the average sound pressure and average vibration velocity of the metamaterial surface. The surface acoustic impedance $Z_{s}$ of the proposed acoustic metamaterial can be expressed as:

6 $$ Z_{s} = \frac{\left\langle p \right\rangle }{{\left\langle u \right\rangle }} $$

where $\left\langle \cdot \right\rangle$ represents the average over the upper surface of the metamaterial, $p$ and $u$ are the sound pressure and particle vibration velocity on the surface of the metamaterial. The sound absorption coefficient of the metamaterial can be predicted numerically by substituting Eq. (6) into Eq. (1).

## Results and discussion

**Figure 3:**
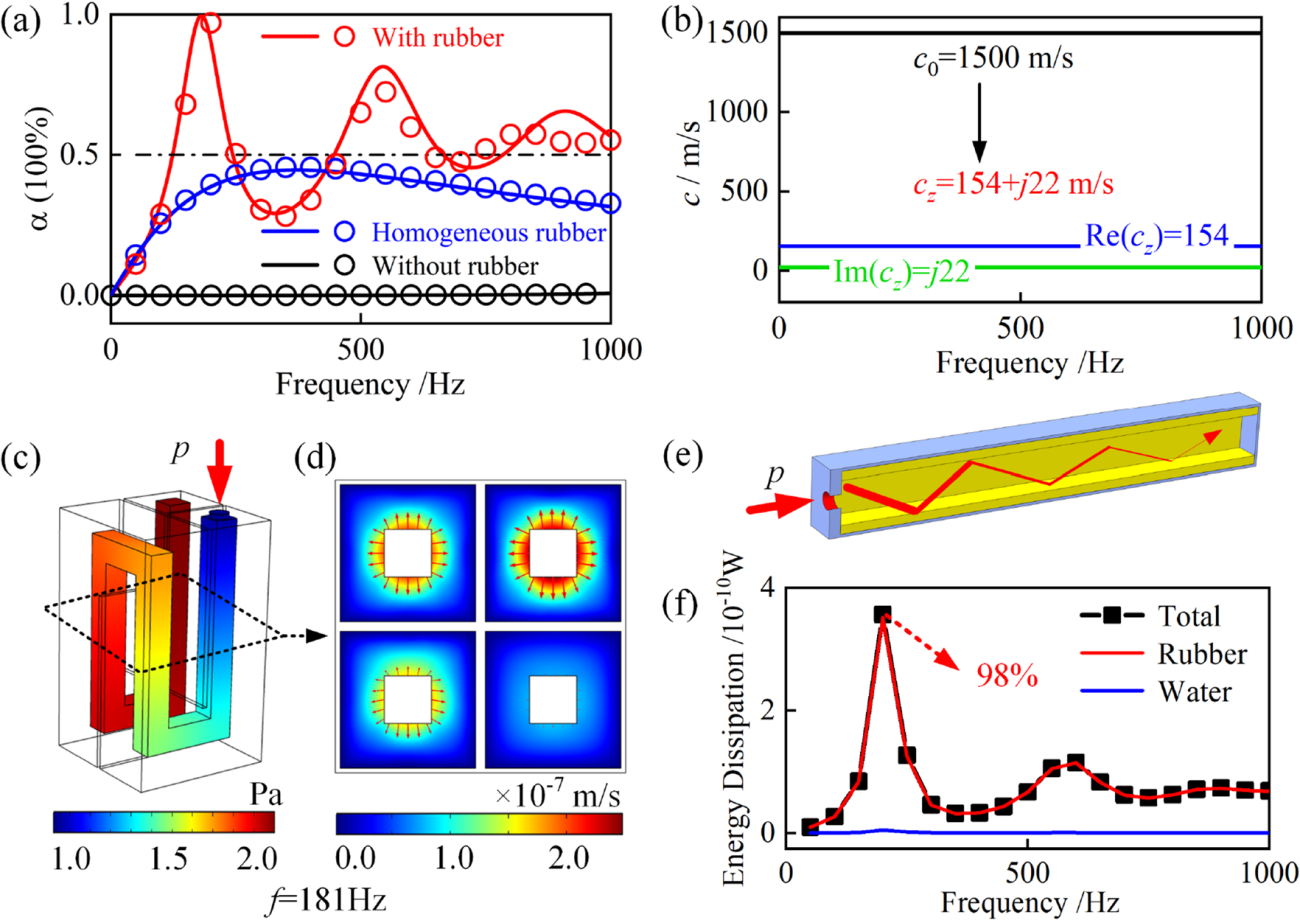
(**a**) Sound absorption coefficients of metamaterials with and without rubber coating and a homogeneous rubber layer of the same thickness. Geometric and material parameters: $d$ = 4 mm, $t_{1}$ = 1 mm, $t_{2}$ = 4.7 mm, $t_{3}$ = 1 mm, $L$ = 30.8 mm, $w$ = 5 mm, $h$ = 50 mm, $L_{eff}$ = 203 mm, $\eta_{r}$ = 0.3, $\rho_{r} = 1100\, {\text{kg/m}}^{{3}}$, $E_{r}$ = 10 MPa, $\mu_{r}$ = 0.49, $\rho_{0} = 1000\,{\text{kg/m}}^{{3}}$ and $c_{0} = {1500}$ m/s. Solid lines and circles represent theoretical and numerical results, respectively. (**b**) The corresponding effective sound speed $c_{z}$ on the water channel, the real part represented by the blue line and the imaginary part represented by the green line. (**c**) Sound pressure distribution at the first resonance frequency of 181 Hz. (**d**) Vibration velocity distribution of the rubber coating on the cross-section of the metamaterial at 181 Hz. The red arrows indicate the vibration velocity distribution on the water channel boundary, the length represents the magnitude of the velocity and the directions represents the direction of the vibration velocity. (**e**) Slow-sound propagation with inherent dissipation on the water channel. The thickness of the red line represents the intensity of the sound wave. (**f**) Energy dissipation in different parts of the metamaterial.

Figure 3a shows the consistency between theoretical predictions and numerical simulation results, especially in the low frequency band, demonstrating the accuracy of the theoretical model. As shown in Fig. 3a, the proposed metamaterial achieves perfect sound absorption ($\alpha$ > 0.99) at 181 Hz with a thickness *H* of only 1/162 of the wavelength, showing good sound absorption performance at the deep subwavelength scale. Meanwhile, the metamaterial also has 81% and 65% sound absorption ability at the second resonance frequency of 545 Hz and third resonance frequency of 910 Hz. The half-absorption bandwidth reaches 553 Hz in the range of [0, 1000 Hz], showing broadband sound absorption capability. As a comparison, the sound absorption coefficients of the metamaterial without rubber coating and the homogeneous rubber of the same thickness are also shown in Fig. 3a. Without the rubber coating on the water channel, the metamaterial has almost no sound absorption capacity, which indicates the importance of rubber coating. In addition, the proposed metamaterial exhibits better low-frequency sound absorption performance than the traditional homogeneous rubber.

Without rubber coating, the effective sound speed $c_{z}$ in the water channel should be the sound speed of water $c_{0}$ = 1500 m/s. After adding the rubber coating on the water channel boundary, it is found that the effective sound speed $c_{z}$ changes. The effective sound speed $c_{z}$ is calculated by Eq. (5), as shown in Fig. 3b. In the studied frequency range, the effective sound speed $c_{z}$ is a constant. The real part of the effective sound speed ${\text{Re}} (c_{z} )$ is 154 m/s, and the imaginary part of the effective sound speed ${\text{Im}} (c_{z} )$ is 22 m/s, in which the real part of the effective sound speed ${\text{Re}} (c_{z} )$ is about 1/10 of the sound speed of water $c_{0}$ = 1500 m/s. The significant decrease of the effective sound speed indicates the phenomenon of slow-sound propagation in the water channel. The phenomenon of slow-sound propagation is usually produced by the strong interaction between sound wave and structures^40–42^. Here, the acoustic impedance of rubber is close to the characteristic impedance of water and has good damping properties. Therefore, rubber is suitable for causing strong interactions and producing slow-sound propagation in the water channel.

The results of numerical simulations are then utilized to explain the slow-sound propagation phenomenon. Figure 3c shows the sound pressure distribution at 181 Hz, and Fig. 3d shows the vibration velocity distribution of the rubber coating on the cross section of the metamaterial. When the sound wave propagates along the water channel, the sound pressure will excite the vibration of the rubber coating. The higher the sound pressure, the stronger the vibration of the rubber coating. As shown in Figs. 3c,d, the sound pressure increases gradually along the water channel, from 1 to 2 Pa, and the vibration velocity of the rubber coating also has the same trend, increasing from $10^{ - 9}$ to $10^{ - 7}$ m/s. Meanwhile, the red arrows representing the vibration velocity on the water channel boundary are perpendicular to the water channel boundary. This indicates that the main vibration mode of the rubber coating is compression vibration, and the shear vibration is weak. Therefore, it is reasonable to consider only the compression vibration of the rubber coating in the theoretical model. The compression vibration of the rubber coating will in turn change the direction of the sound wave, causing the refraction of the sound wave, as shown in Fig. 3e. The refraction of the sound wave increases the propagation path of the sound wave, that is, the effective sound speed decreases, resulting in the phenomenon of slow-sound propagation. Also, the damping properties of rubber brings energy dissipation during the compression vibration of the rubber coating. Therefore, the sound energy can be absorbed during the sound wave propagation in the water channel. As shown in Fig. 3f, the energy dissipation of the whole metamaterial, rubber and water are plotted, respectively. The energy dissipation of rubber accounts for 98% of the total energy dissipation, while the energy dissipation of water only accounts for 2%. Therefore, the vibration of the rubber coating plays a major role in the dissipation of sound energy. The introduction of rubber coating on the water channel boundary results in the inherently dissipative slow-sound propagation in the water channel, which is the key to achieving perfect sound absorption at low frequencies.

**Figure 4:**
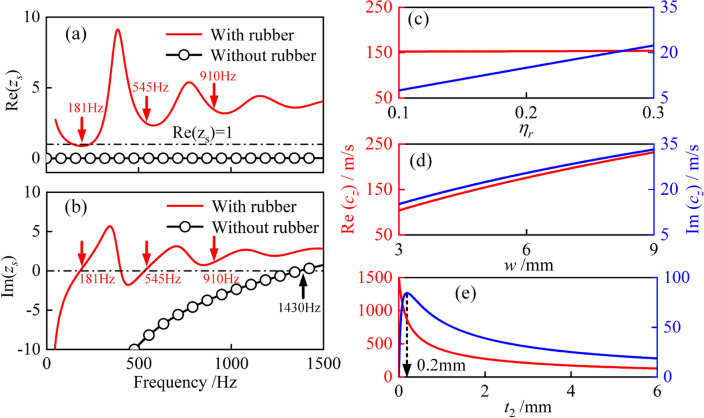
(**a**,**b**) Normalized surface acoustic resistance ${\text{Re}} \left( {z_{s} } \right)$ and reactance ${\text{Im}} \left( {z_{s} } \right)$ of the metamaterial with and without rubber coating, where $z_{s} = Z_{s} /Z_{0}$. The solid lines and circles represent theoretical predictions and finite element (FE) simulation results, respectively. Colored arrows indicate the absorption peak frequencies. (**c**–**e**) The effective sound speed $c_{z}$ on the water channel with different isotropic loss factor $\eta_{r}$, channel width $w$ and the thickness of rubber coating $t_{2}$. Other parameters are consistent with those in Fig. 2a. The red solid lines represent the real part of $c_{z}$, and the blue solid lines represent the imaginary part of $c_{z}$.

From the perspective of acoustic impedance, the mechanism of sound absorption and the influence of slow-sound propagation with inherent dissipation are investigated. When inherently dissipative slow-sound propagation occurs, the effective sound speed $c_{z}$ becomes 154 + *j*22 m/s. According to Eq. (3), the increase of ${\text{Im}} (c_{z} )$ from 0 to 22 m/s, will directly increase the acoustic resistance of the water channel, which will ultimately increase the surface acoustic resistance of the metamaterial. This can be seen from the comparison of the normalized surface acoustic resistance of metamaterial with and without rubber coating, as shown in Fig. 4a. Without the rubber coating, the normalized surface acoustic resistance is close to 0. After adding the rubber coating, the normalized surface acoustic resistance is greatly improved. At 181 Hz, the normalized surface acoustic resistance reaches 1, which is the premise for perfect sound absorption. According to Eq. (3), the decrease of ${\text{Re}} (c_{z} )$ from 1500 to 154 m/s, will directly improve the acoustic reactance of the water channel, and finally improve the surface acoustic reactance of the metamaterial. This can be seen from the comparison of the normalized surface acoustic reactance of metamaterials with and without rubber coating, as shown in Fig. 4b. Without the rubber coating, the normalized surface acoustic reactance is low, and the corresponding resonance frequency is 1430 Hz. After adding the rubber coating, the normalized surface acoustic reactance is greatly increased, and the resonance frequency is reduced to 181 Hz, which is the premise to achieve low-frequency sound absorption. At 181 Hz, the normalized surface acoustic resistance is 1 and the normalized surface acoustic reactance is 0 at the same time. The metamaterial meets the impedance matching condition and achieves perfect low-frequency sound absorption. Besides, at 545 Hz and 910 Hz, the normalized surface acoustic resistance is slightly greater than 1, and the normalized surface acoustic reactance reaches a local minimum. Therefore, the metamaterial also has sound absorption coefficients of 0.81 and 0.65 respectively, which helps to broaden the half-absorption bandwidth. In general, the slow-sound propagation with inherent dissipation on the water channel can reduce the resonant frequency and provide effective acoustic resistance to the metamaterial.

The slow-sound propagation with inherent dissipation is the key to achieving low frequency perfect sound absorption, so it is necessary to explore the influence of key parameters to the effective sound speed $c_{z}$. Figure 4c shows the relationship between the effective sound speed $c_{z}$ and the rubber loss factor $\eta_{r}$. The range of $\eta_{r}$ is [0.1, 0.3], which is suitable for rubber. As $\eta_{r}$ increases, the real part of the effective sound speed ${\text{Re}} (c_{z} )$ remains almost unchanged, while the imaginary part of the effective sound speed ${\text{Im}} (c_{z} )$ increases linearly, from 7 to 22 m/s. According to Eq. (3), the increase of the effective sound speed ${\text{Im}} (c_{z} )$ will increase the acoustic resistance of the metamaterial. Therefore, the rubber loss factor $\eta_{r}$ is beneficial to increase the acoustic resistance of the metamaterial. Figure 4d shows the relation between the effective sound speed $c_{z}$ and channel width $w$. The increase of channel width $w$ is directly proportional to the real part and imaginary part of the effective sound speed $c_{z}$. The ${\text{Re}} (c_{z} )$ increases from 105 to 230 m/s and the ${\text{Im}} (c_{z} )$ increases from 15 to 33 m/s. Figure 4e shows the relation between the effective sound speed $c_{z}$ and rubber coating thickness $t_{2}$. The increase of $t_{2}$ is inversely proportional to ${\text{Re}} (c_{z} )$. When the thickness of rubber coating is less than 2 mm, the ${\text{Re}} (c_{z} )$ decreases rapidly from 1500 to 270 m/s. When the thickness of rubber coating is larger than 2 mm, the ${\text{Re}} (c_{z} )$ decreases slowly from 270 to 120 m/s. The imaginary part of the effective sound speed ${\text{Im}} (c_{z} )$ first increases rapidly to 84 m/s when the thickness of rubber coating increases from 0 to 0.2 mm, and then gradually decreases to 19 m/s as the thickness of rubber coating increases to 6 mm. The effective sound speed can be adjusted as needed to achieve the tunable absorption performance of this metamaterial.

**Figure 5:**
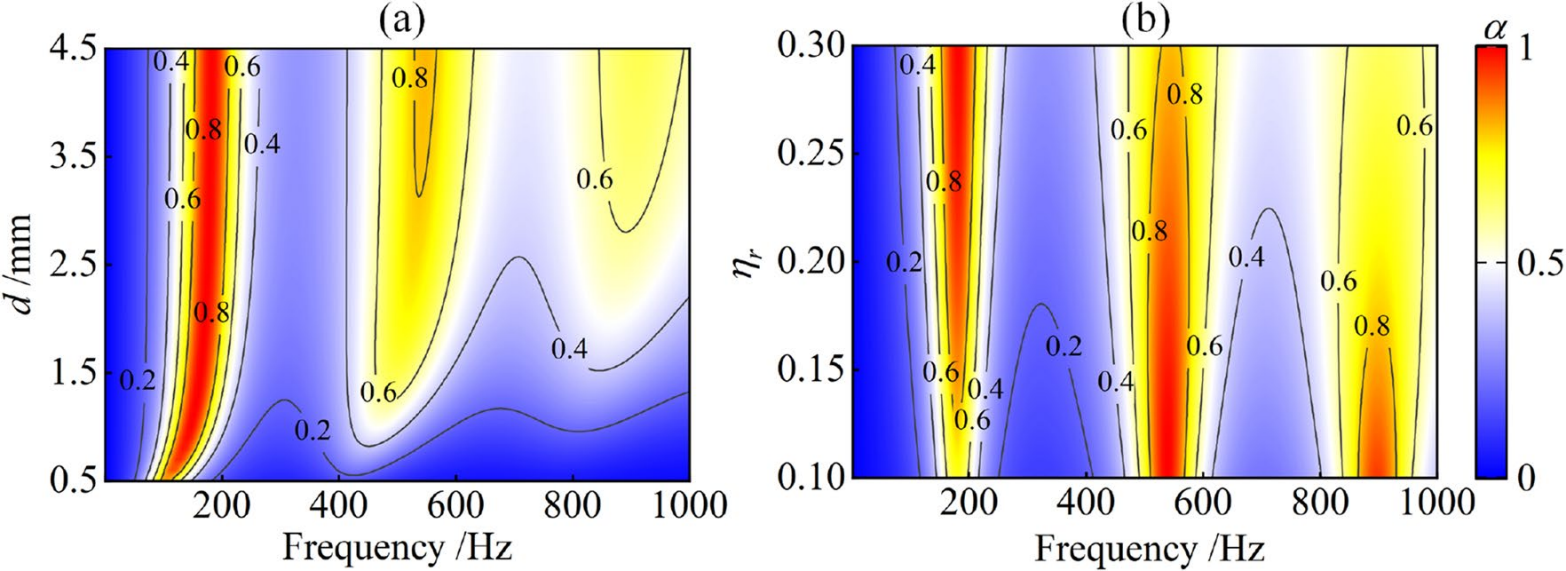
Sound absorption coefficient of the proposed metamaterial: (**a**) effect of perforation diameter $d$; (**b**) effect of rubber loss factor $\eta_{r}$.

The sound absorption performance of the proposed metamaterial is closely related to its structural and material parameters. The perforation of the proposed metamaterial allows the incident sound waves to enter the space-coiled channel with rubber coating, causing slow-sound propagation and achieving low-frequency sound absorption. Figure 5a shows the effect of the perforation diameter $d$ on the sound absorption coefficient. As the perforation diameter decreases, the sound absorption peaks shift slightly to lower frequencies and the peak values decrease, This is because, according to Eq. (2), the reduction of perforation diameter $d$ will reduce the perforation ratio and increase the surface acoustic reactance ${\text{Im}} \left( {z_{s} } \right)$, resulting in the zero point in Fig. 4b shifting to the left and the resonant frequency moving to lower frequencies. In addition, the change of perforation diameter has a greater effect on the second and third sound absorption peaks. Figure 5b shows the effect of the rubber loss factor $\eta_{r}$ on the sound absorption coefficient. As the loss factor $\eta_{r}$ increases from 0.1 to 0.3, the value of the first absorption peak increases and the second and third absorption peaks decrease, while the frequencies of the absorption peaks remain unchanged. The different changes of the different absorption peaks can be explained by the trend in Fig. 4c. As shown in Fig. 4c, as the loss factor increases, the imaginary part ${\text{Im}} \left( {c_{z} } \right)$ of the effective sound speed gradually increases. According to Eq. (3), the acoustic resistance of the water channel increases, and ultimately the surface acoustic resistance of the metamaterial increases. This means that an increase in the rubber loss factor can increase the acoustic resistance of the metamaterial. With this trend, as the loss factor increases to 0.3, the surface acoustic resistance ${\text{Re}} \left( {z_{s} } \right)$ at the three resonant frequencies is shown in Fig. 4a. In other words, as the loss factor increases in the range [0.1, 0.3], the surface acoustic resistance ${\text{Re}} \left( {z_{s} } \right)$ gradually approaches 1 at the first resonant frequency of 181 Hz, and gradually moves away from 1 at the second and third resonant frequencies of 545 Hz and 910 Hz. Therefore, the first absorption peak increases, but the second and third absorption peaks decrease.

**Figure 6:**
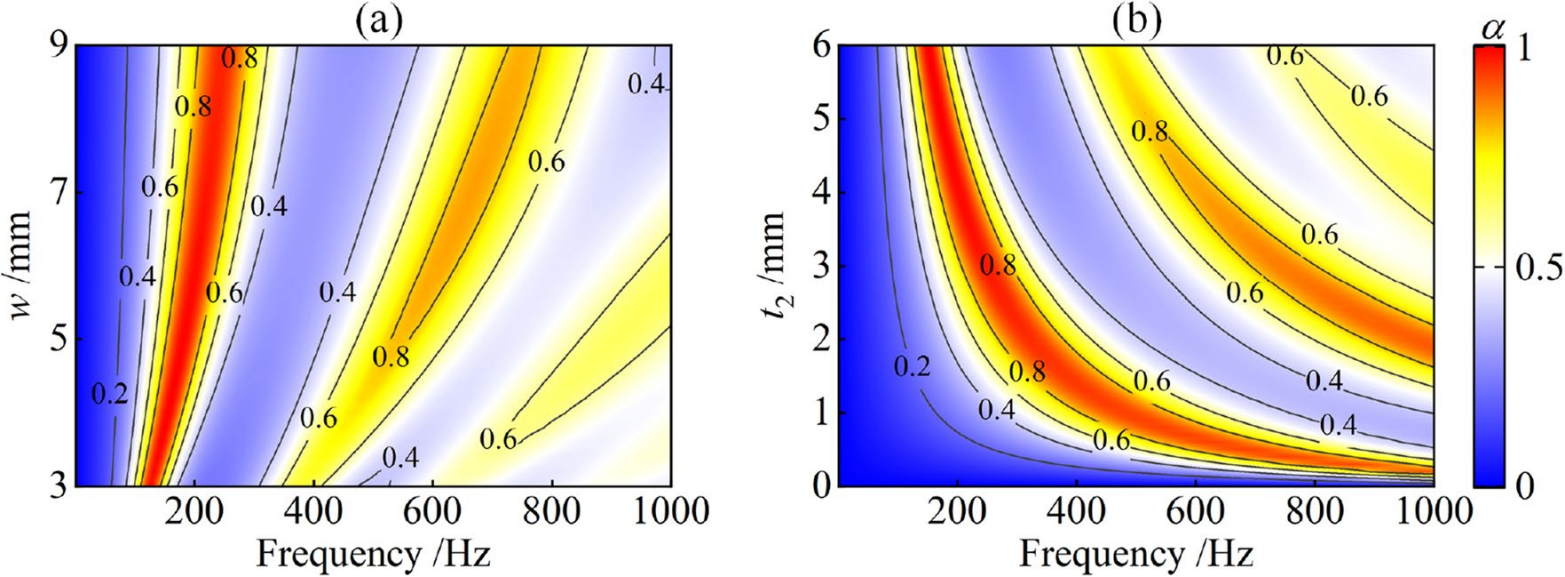
Sound absorption coefficient of the proposed metamaterial: (**a**) effect of the width of the space-coiled water channel $w$; (**b**) effect of the thickness of rubber coating $t_{2}$.

Figure 6a shows the effect of the space-coiled water channel width $w$ on the sound absorption coefficient of the proposed metamaterial. As the channel width $w$ increases, the sound absorption peaks shift to higher frequencies, which can be explained by the trend in Fig. 4d. As shown in Fig. 4d, the real part of the effective sound speed ${\text{Re}} (c_{z} )$ gradually increases, causing a corresponding decrease in the acoustic reactance of the water channel according to Eq. (3), which eventually decreases the surface acoustic reactance of the metamaterial and results in a shift of the absorption peak to higher frequencies. Figure 6b shows the effect of the rubber coating thickness $t_{2}$ on the sound absorption coefficient. With the increase of the rubber thickness $t_{2}$, the sound absorption peak gradually moves towards lower frequencies, which can be explained by the trend in Fig. 4e. As shown in Fig. 4e, the real part of the effective sound speed ${\text{Re}} (c_{z} )$ gradually decreases with increasing rubber thickness $t_{2}$, resulting in an increase in the acoustic reactance of the water channel according to Eq. (3), which eventually increases the surface acoustic reactance of the metamaterial and causes a shift of the absorption peak to lower frequencies.

**Figure 7:**
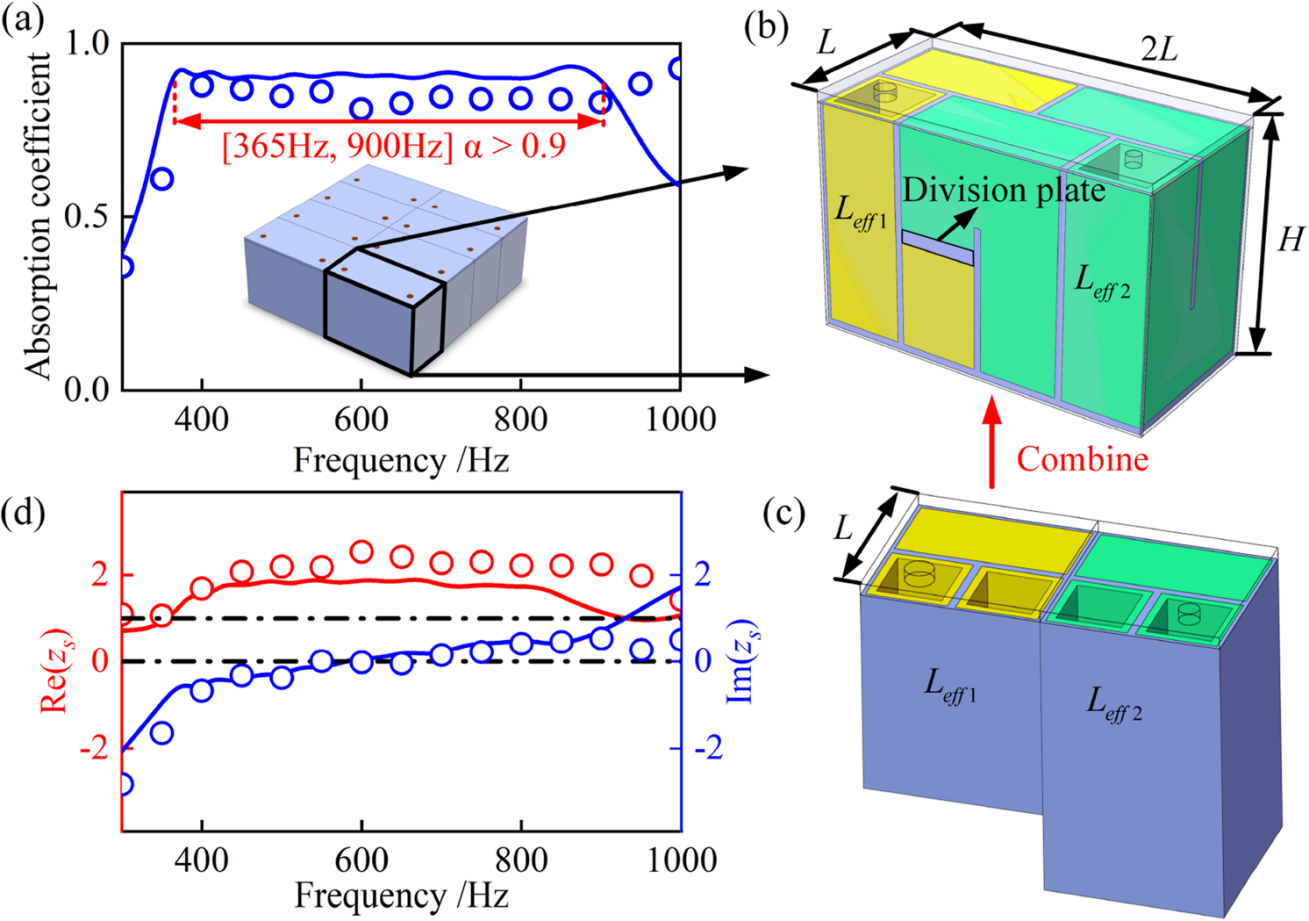
(**a**–**c**) Broadband sound absorption of the ultrathin acoustic metamaterial with eight parallel hybrid units. Each hybrid unit is composed of a metamaterial unit with higher height and a metamaterial unit with lower height. A division plate is set to separate the two units. The blue line and circles represent the sound absorption coefficient from theoretical predictions and FE simulation results, respectively. (**d**) The normalized surface acoustic resistance (red) and acoustic reactance (blue) of the ultra-broadband sound absorber.

By tailoring the key parameters of the proposed metamaterial, an ultra-broadband sound absorber is designed, which can achieve perfect sound absorption ($\alpha > 0.9$) from 365 to 900 Hz, as shown in Fig. 7a (see the Supplementary materials for geometric parameters). The smallest periodic unit of the ultra-broadband sound absorber consists of 8 hybrid units with customized parameters in parallel. The customized parameters are the thickness of rubber coating $t_{2}$, channel width $w$, isotropic loss factor $\eta_{r}$, perforation diameter $d$ and channel height $h$. Other parameters, including panel thickness $t_{1}$, wall thickness $t_{3}$, and unit width $L$ remain unchanged. The hybrid unit is designed to further reduce the thickness. Each hybrid unit is composed of a metamaterial unit with higher height and a metamaterial unit with lower height, as shown in Fig. 7b,c. The thickness of the division plate is adjusted to ensure that the effective channel length $L_{eff1}$ and $L_{eff2}$ of these two units remains unchanged after the combination. By carefully tailoring, the thickness $H$ is reduced to 33 mm, showing ultra-broadband low-frequency sound absorption at the deep subwavelength scale. The normalized surface acoustic impedance of the ultra-broadband sound absorber can be expressed as $z_{s} = {{16\left( {\sum\limits_{m = 1}^{16} {{1 \mathord{\left/ {\vphantom {1 {Z_{m} }}} \right. \kern-0pt} {Z_{m} }}} } \right)^{ - 1} } \mathord{\left/ {\vphantom {{16\left( {\sum\limits_{m = 1}^{16} {{1 \mathord{\left/ {\vphantom {1 {Z_{m} }}} \right. \kern-0pt} {Z_{m} }}} } \right)^{ - 1} } {\left( {\rho_{0} c_{0} } \right)}}} \right. \kern-0pt} {\left( {\rho_{0} c_{0} } \right)}}$, where $Z_{m}$ is the surface acoustic impedance of the $m$th metamaterial unit. As shown in Fig. 7d, the normalized surface acoustic resistance is around 1 and the normalized surface acoustic reactance is around 0, so the sound absorption coefficient can reach 0.9 at the range of [365–900 Hz].

## Conclusions

In this work, an ultrathin acoustic metamaterial is proposed for broadband low-frequency underwater sound absorption with deep subwavelength thickness. The existence of the slow-sound propagation phenomenon is proved by theoretical calculation of the effective sound speed. Through the combination of numerical simulation and theoretical calculation, the sound absorption mechanism analysis finds that the rubber coating bounded to the wall of the space-coiled water channel leads to the slow-sound propagation with inherent dissipation, which is the key to satisfy the impedance matching condition and achieve perfect low-frequency sound absorption. The relationship between the effective sound speed and the parameters of the sound absorber is discussed in detail, which demonstrates the tunability of the sound absorption performance of this metamaterial. By tailoring the key parameters and careful structural design, an ultra-broadband sound absorber with eight parallel hybrid units is constructed, and its thickness is further reduced by combining two metamaterial units of different heights into one hybrid unit. This work is of great significance to the design of underwater acoustic metamaterial and the control of underwater acoustic waves.

See the Supplementary materials for the details of the complete finite element model considering steel parts as elastic bodies, the validation of the acoustically rigid steel assumption, and the geometric parameters of the proposed acoustic metamaterial.

## Supplementary Information

Supplementary Information.

## Data Availability

The data that support the findings of this study are available from the corresponding author upon reasonable request.
