# Supplementary material for: Ultrathin acoustic metamaterial as super absorber for broadband low-frequency underwater sound
Source: Sci Rep. 2023 May 17;13:7983. doi: 10.1038/s41598-023-34993-0 (PMC10192347; doi:10.1038/s41598-023-34993-0)
Supplement: Supplementary file 1 — Supplementary Information. [file 41598_2023_34993_MOESM1_ESM.pdf]

Supplementary materials for "Ultrathin acoustic metamaterial as super absorber for broadband low-frequency underwater sound"

Xindong Zhou[1,2], Xiaochen Wang[1,2], and Fengxian Xin[1,2,*]

[1]*State Key Laboratory for Strength and Vibration of Mechanical Structures, Xi'an Jiaotong University, Xi'an 710049, P.R. China*

[2]*MOE Key Laboratory for Multifunctional Materials and Structures, Xi'an Jiaotong University, Xi'an 710049, P.R. China*

Section 1: Validation of the assumption of rigidity of steel parts

In order to prove the assumption that all steel parts in the theoretical model are acoustically rigid, a complete finite element (FE) model is established that treats steel parts as elastic bodies , as shown in Fig. S1. In the complete FE model, the solid mechanics module is selected for the rubber region and steel region displayed in yellow and blue respectively, and the thermo-viscous acoustic module is selected for the water region displayed in green. An additional water region is set above the metamaterial surface to simulate the acoustic incident field, and plane wave incidence is set on the top surface of the acoustic incident field. Due to the continuity of sound pressure and particle vibration velocity at the water-rubber, water-steel and rubber-steel coupling interfaces, the acoustic-structure interaction boundaries are set at these coupling interfaces. The interfaces between rubber and steel are set to the same displacement constraints as the relative displacement of the two is zero. Periodic boundary conditions are applied to model this periodic acoustic metamaterial. The bottom surface of the metamaterial is set to a fixed constraint in consideration of rigid backing. The material properties of the steel are selected as: density $\rho_s = 7850\ \text{kg/m}^3$, Young's modulus E_s =205GPa, Poisson's ratio $\mu_s = 0.3$.

*fengxian.xin@gmail.com or fxxin@mail.xjtu.edu.cn (F.X. Xin)

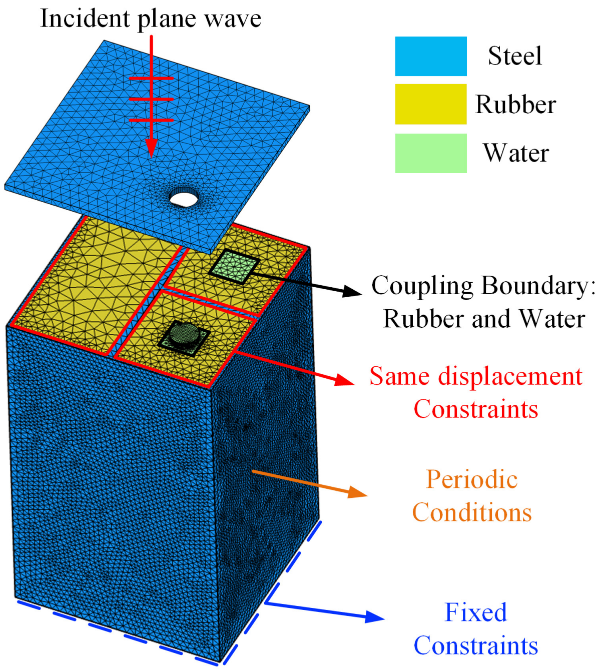

Fig. S1. Complete finite element model for the sound absorption of the proposed metamaterial. The blue, yellow and green sections represent the steel, rubber and water areas, respectively.

Based on the complete FE model, numerical sound absorption coefficients considering that take into account the elastic behaviour of the steel parts can be obtained and compared with the theoretical model and the rigid FE model in the paper, as shown in Fig. S2. The results of the two numerical simulations agree well, and the differences between the two are relatively small and negligible due to the coupling vibration of the steel plate and the rubber . In addition, both numerical simulations are consistent with the theoretical results. This indicates that the assumption of acoustically rigid steel is feasible and that the rigid FE model is sufficiently accurate for modeling the proposed metamaterial.

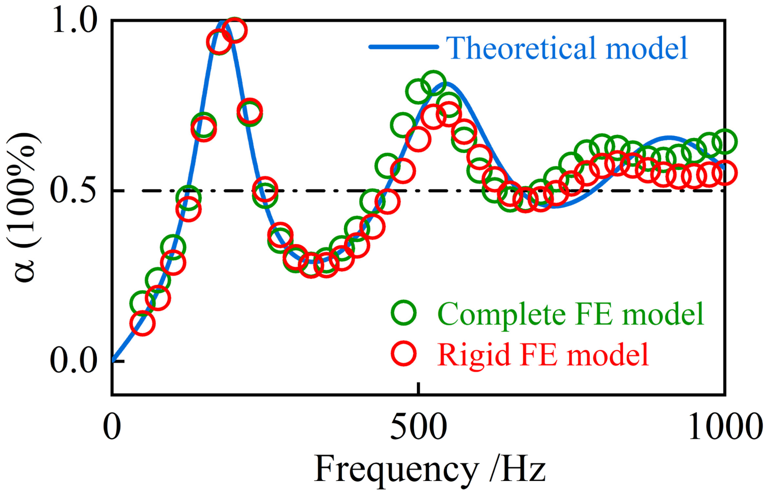

Fig. S2. Sound absorption coefficients of the theoretical model (blue curve), the complete FE model considering the elastic behavior of the steel parts (green circles), and the rigid FE model based on the assumption of acoustically rigid steel parts (red circles) of the proposed metamaterial.

To demonstrate that the coupling effect between rubber and steel has been taken into account , the vibration velocity distributions at the three resonant frequencies (i.e., 181Hz, 545Hz and 910Hz) in the cross section shown in Fig. S3(a) are presented in Fig. S3(b-d). In particular, the vibration velocities at the rubber-steel coupling interfaces are indicated by arrows, where the red and gray arrows represent the vibration velocities at the rubber and steel boundaries, respectively. As can be seen from Fig. S3(b-d), the vibration velocities of rubber and steel at the coupling interfaces are the same in both magnitude and direction. This indicates that the coupling effect between rubber and steel has been well taken into account.

Moreover, the strength of the rubber-steel coupling vibration can explain the slight difference in the results of the two finite element models in Fig. S2. As shown in Fig. S3(b), taking the coupling vibration of surrounding steel and rubber as an example, the coupling vibration velocity is mainly along the y-direction and the amplitude is small, so that the results of the two finite element models are basically consistent at 181Hz. Noted that in Fig. S3(b-d), the colour legends have different colour ranges, while the red and grey arrows have the same length scale factor. As shown in Fig. S3(c), the coupling vibration velocity is mainly along the x-direction and the amplitude increases at 545Hz. Furthermore, as shown in Fig. S3(d), the coupling vibration velocity increases along the y-direction at 910Hz. Therefore, a slight difference between the elastic FE model and the rigid FE model occurs around the resonant frequencies of 545Hz and the 910Hz. However, in the frequency range considered, the finite element model based on the acoustically rigid steel assumption still predicts the sound absorption performance of the proposed acoustic metamaterial well even in the presence of coupled rubber-steel vibrations.

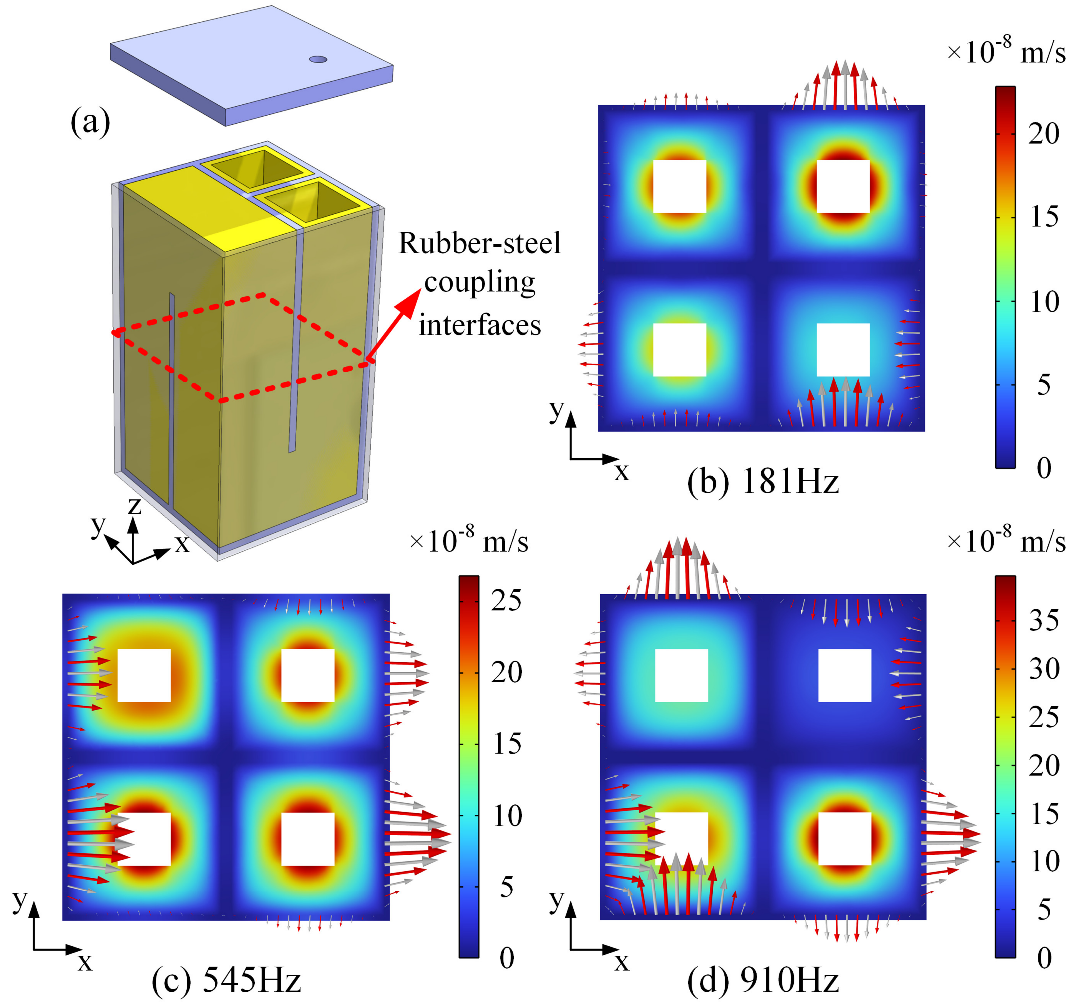

Fig. S3. (a) Geometric model of the cross-section of the proposed metamaterial and rubber-steel coupling interface. (b-d) Distribution of vibration velocities at three resonant frequencies (i.e., 181Hz, 545Hz and 910Hz) in the cross-section, with red and gray arrows indicating the vibration velocities at the rubber and steel boundaries, respectively. The colour legends have different colour ranges, while the red and grey arrows in (b-d) have the same length scale factor.

Section 2: Geometric parameters of the ultra-broadband sound absorber

The detailed geometric parameters of the ultra-broadband sound absorber are listed in Tables. S1. There are eight hybrid units and each hybrid unit is constructed by two metamaterial units. The h_b in Table. S1 represents the channel height before combining. After the combination, the channel heights of the hybrid units h_a are all 32mm. The key parameters determined the external shape of the absorber, i.e. t_1, t_3, L and h_a, are constant.

Table. S1. Geometric parameters of the ultra-broadband sound absorber

**Absorber**	**Unit**	d **(mm)**	t_1 **(mm)**	t_2 **(mm)**	t_3 **(mm)**	w **(mm)**	L **(mm)**	η_r	h_b **(mm)**	L_{eff} **(mm)**

**Hybrid unit 1**	Unit 1	3.17	1.00	2.16	1.00	7.18	25.00	0.13	17.00	71.00
	Unit 2	3.18	1.00	2.87	1.00	5.76	25.00	0.13	45.49	185.00
**Hybrid unit 2**	Unit 3	3.02	1.00	1.61	1.00	8.28	25.00	0.11	19.82	82.30
	Unit 4	2.74	1.00	1.37	1.00	8.76	25.00	0.29	40.04	163.20
**Hybrid unit 3**	Unit 5	2.59	1.00	1.77	1.00	7.96	25.00	0.15	21.03	87.10
	Unit 6	1.96	1.00	1.40	1.00	8.70	25.00	0.14	39.28	160.10
**Hybrid unit 4**	Unit 7	2.58	1.00	2.00	1.00	7.50	25.00	0.26	21.64	89.60
	Unit 8	3.52	1.00	2.43	1.00	6.64	25.00	0.12	38.81	158.20
**Hybrid unit 5**	Unit 9	3.81	1.00	1.61	1.00	8.28	25.00	0.21	22.41	92.60
	Unit 10	2.22	1.00	1.98	1.00	7.54	25.00	0.22	38.09	155.40
**Hybrid unit 6**	Unit 11	2.25	1.00	1.78	1.00	7.94	25.00	0.17	22.42	92.70
	Unit 12	3.16	1.00	2.87	1.00	5.76	25.00	0.21	36.34	148.40
**Hybrid unit 7**	Unit 13	2.10	1.00	2.34	1.00	6.82	25.00	0.20	30.30	124.20
	Unit 14	3.04	1.00	1.88	1.00	7.74	25.00	0.27	31.95	130.80
**Hybrid unit 8**	Unit 15	3.33	1.00	3.25	1.00	5.00	25.00	0.20	30.56	125.20
	Unit 16	2.73	1.00	1.56	1.00	8.38	25.00	0.17	31.34	128.40
